# Knowledge, Attitudes, and Practices of Small Ruminant and Pig Farmers Regarding Tsetse Flies and Animal African Trypanosomosis in Southern Benin

**DOI:** 10.1155/vmi/8578242

**Published:** 2026-09-30

**Authors:** Zul-Kifl Affolabi, Razaki A. Ossè, Minassou Juvénal Ahouandjinou, Prudenciène A. Agboho, Koffi Djigbodi Koumodji, Steve Zinsou Hougbe, Côme Zinsou Koukpo, Ehouzou Olivier Oussou, Arsène Jacques Fassinou, Casimir Kpanou, Filémon Tokponnon, Germain Gil Padonou, Martin Akogbeto

**Affiliations:** ^1^ Laboratoire des Sciences Animales et Halieutiques, Unité de Recherche en Santé Animale et Biosécurité Université Nationale d’Agriculture (UNA) Kétou Benin; ^2^ École de Gestion et d’Exploitation des Systèmes d’Elevage (EGESE) Université Nationale d’Agriculture (UNA) Kétou Benin; ^3^ Centre de Recherche Entomologique de Cotonou (CREC) Cotonou Benin; ^4^ Centre International de Recherche-Développement sur l’Elevage en Zone Subhumide (CIRDES) Bobo-Dioulasso Burkina Faso; ^5^ Laboratoire de Biologie et de Typage Moléculaire en Microbiologie (LBTMM) Département de Biochimie et de Biologie Cellulaire (BBC) Université d’Abomey-Calavi (UAC) Abomey-Calavi Benin uac.bj; ^6^ Faculté des Sciences et Techniques (FAST) Université d’Abomey-Calavi (UAC) Abomey-Calavi Benin uac.bj; ^7^ Institut Supérieur des Sciences et de Médecine Vétérinaire (ISSMV) Dalaba Guinea; ^8^ École Polytechnique d’Abomey-Calavi (EPAC) Université d’Abomey-Calavi Abomey-Calavi Benin uac.bj

**Keywords:** animal African trypanosomosis, Benin, *Glossina* spp., livestock farmers, tsetse flies, vector control

## Abstract

**Background:**

Animal African trypanosomosis (AAT) remains a major constraint to livestock production across sub‐Saharan Africa despite ongoing control efforts. Although several studies have assessed farmers’ knowledge, attitudes, and practices (KAP) regarding AAT in cattle production systems, little is known about small ruminant and pig farmers, particularly in Benin. This study assessed farmers’ KAP regarding tsetse flies and AAT and identified the sociodemographic factors associated with disease awareness in southern Benin.

**Methods:**

A community‐based cross‐sectional survey was conducted between September and December 2024 among 384 randomly selected goat, sheep, and pig farmers from six communes in southern Benin. Data were collected using a structured, pretested questionnaire administered through the KoboCollect platform. Descriptive statistics were used to summarize respondents’ characteristics and KAP outcomes. Associations between sociodemographic variables and knowledge were assessed using chi‐square tests, while multivariable logistic regression identified independent predictors of knowledge. The results are presented as odds ratios (ORs) with 95% confidence intervals (95% CIs). Statistical significance was set at *p* < 0.05.

**Results:**

Among the 384 respondents, 62.24% were men, 45.57% were aged 41–50 years, and 56.77% had no formal education. Although most farmers (83.59%) correctly recognized tsetse flies, only 16.93% identified them as vectors of animal trypanosomosis, and 81.3% had never heard of the disease. Knowledge was significantly associated with sex (*χ* ^2^ = 74.527; *p* < 0.0001), age (*χ* ^2^ = 299.04; *p* < 0.0001), marital status (*χ* ^2^ = 873.37; *p* < 0.0001), educational level (*χ* ^2^ = 219.96; *p* < 0.0001), and occupation (*χ* ^2^ = 214.23; *p* < 0.001). In multivariable analysis, secondary and higher education, male sex, and agricultural occupations remained significant predictors of knowledge. While insecticides were perceived as the most effective control method (44.53%), only 14.1% of respondents reported using them, and two‐thirds implemented no specific vector control measures. Trypanocides were used by only 11.5% of farmers, whereas reliance on traditional plant‐based practices remained uncommon (2.86%).

**Conclusions:**

Small ruminant and pig farmers in southern Benin generally recognize tsetse flies but have limited knowledge of their role in AAT transmission and report low adoption of effective prevention and control measures. These findings highlight the need for targeted awareness campaigns, strengthened veterinary extension services, and integrated One Health approaches to improve AAT prevention and sustainable livestock production in Benin.

## 1. Background

Animal African trypanosomosis (AAT) remains one of the most important vector‐borne diseases limiting livestock production across sub‐Saharan Africa [1]. The disease is caused by several *Trypanosoma* species, principally *Trypanosoma congolense*, *T. vivax*, and *T. brucei* sensu lato and is transmitted cyclically by tsetse flies (*Glossina* spp.) and, for some parasite species, mechanically by other hematophagous flies such as *Stomoxys* spp. and tabanids [2–4]. AAT compromises the health and productivity of domestic animals, particularly cattle, but also sheep, goats, and pigs, causing chronic anemia, weight loss, reproductive failure, reduced milk and meat production, increased mortality, and diminished draft power, thereby imposing substantial socioeconomic losses on livestock‐dependent communities throughout the continent [5, 6]. The disease is responsible for substantial annual economic losses, estimated at approximately US$4.5 billion across sub‐Saharan Africa [7, 8].

Despite decades of research and vector control initiatives, AAT remains endemic in many West African countries, including Benin [9, 10]. Although recent progress has been achieved in controlling Human African trypanosomiasis (HAT), animal trypanosome transmission persists because competent vectors continue to circulate in suitable ecological habitats and domestic animal reservoirs remain widely distributed [11]. Consequently, AAT continues to represent a major constraint to livestock productivity, food security, and rural livelihoods, particularly in smallholder production systems where access to veterinary services is often limited.

The epidemiology of AAT is closely linked to the ecology of its vectors. Tsetse flies are highly sensitive to environmental conditions, with their abundance and spatial distribution being influenced by vegetation cover, humidity, temperature, wildlife reservoirs, water bodies, and livestock density [12]. Southern Benin provides favorable ecological conditions for several hematophagous fly species because of its subequatorial climate, mosaic landscapes of forests and savannahs, extensive hydrographic network, and mixed crop–livestock production systems. In addition to cyclical transmission by *Glossina* spp., the widespread occurrence of mechanically competent biting flies may contribute to maintaining trypanosome circulation among domestic animals, thereby increasing the complexity of disease control [13].

Current AAT control strategies rely principally on vector control, chemotherapy with trypanocidal drugs, improved animal husbandry, strategic surveillance, and the promotion of trypanotolerant livestock breeds [14]. However, the long‐term effectiveness of these interventions depends largely on farmers’ knowledge of disease transmission, their perception of disease risk, and their willingness to adopt appropriate prevention and control measures. Knowledge, attitudes, and practices (KAP) surveys therefore constitute valuable tools for identifying behavioral barriers, misconceptions, and priority areas for extension programs. Such information enables veterinary authorities to develop evidence‐based interventions that are better adapted to local epidemiological and socioeconomic contexts [15].

Previous KAP studies conducted in several African countries have reported variable levels of awareness regarding tsetse flies and AAT [16, 17]. While many livestock farmers are able to recognize tsetse flies based on their appearance or biting behavior, considerably fewer correctly understand their role in parasite transmission or consistently implement recommended vector control measures [16]. Low educational attainment, limited veterinary extension services, restricted access to animal health information, and financial constraints have frequently been identified as important determinants of poor knowledge and suboptimal disease management [14, 17]. Nevertheless, substantial heterogeneity exists among countries and livestock production systems, emphasizing the need for locally generated evidence to guide context‐specific control strategies.

In Benin, most research has focused on the epidemiology of trypanosome infections, vector ecology, and disease prevalence in cattle [10]. Comparatively little attention has been paid to small ruminants and pigs, despite their important contribution to household income, food security, and livelihood resilience [18, 19]. Indigenous breeds such as Djallonké sheep and West African Dwarf goats exhibit varying degrees of trypanotolerance, yet they remain susceptible to productivity losses associated with trypanosome infections [20, 21]. Similarly, pigs can serve as important hosts for animal trypanosomes and may contribute to maintaining parasite transmission within endemic areas. Despite their epidemiological and economic importance, these livestock species remain underrepresented in awareness programs and disease control initiatives.

Furthermore, although the World Health Organization validated the elimination of HAT as a public health problem in Benin in 2022, this achievement should not be interpreted as the disappearance of trypanosome transmission [11]. The continued presence of tsetse flies and mechanically transmitting biting flies, together with persistent circulation of animal trypanosomes, highlights the importance of maintaining surveillance from a One Health perspective that recognizes the close interconnections among animal health, human health, and the environment.

Although several studies have evaluated farmers’ KAP regarding AAT in cattle production systems across sub‐Saharan Africa [16, 22, 23], comparable evidence remains scarce for small ruminant and pig farming systems, particularly in Benin. To our knowledge, no previous study has comprehensively assessed farmers’ knowledge of tsetse flies and AAT among these livestock sectors while simultaneously examining the sociodemographic determinants of disease awareness in the post‐HAT elimination context. This knowledge gap limits the development of targeted awareness campaigns, veterinary extension programs, and integrated vector management strategies specifically adapted to smallholder farmers.

Therefore, the present study aimed to assess the KAP of small ruminant and pig farmers regarding tsetse flies and AAT in southern Benin and to identify the sociodemographic factors associated with disease awareness. By generating baseline evidence from previously understudied livestock sectors, this study provides information that can support the design of context‐specific control strategies and contribute to strengthening One Health approaches for the sustainable management of animal trypanosomosis in Benin.

## 2. Materials and Methods

### 2.1. Study Area

The study was conducted between September and December 2024 in six communes of southern Benin: Allada (Atlantique Department), Bopa (Mono Department), Dangbo (Ouémé Department), Djidja (Zou Department), Dogbo (Couffo Department), and Kétou (Plateau Department) (Figure 1). These communes were purposively selected because they present ecological conditions favorable for tsetse flies and other hematophagous vectors, including the presence of permanent or seasonal water bodies, forest remnants, wetlands, grazing areas, and relatively high densities of domestic livestock.

**FIGURE 1:**
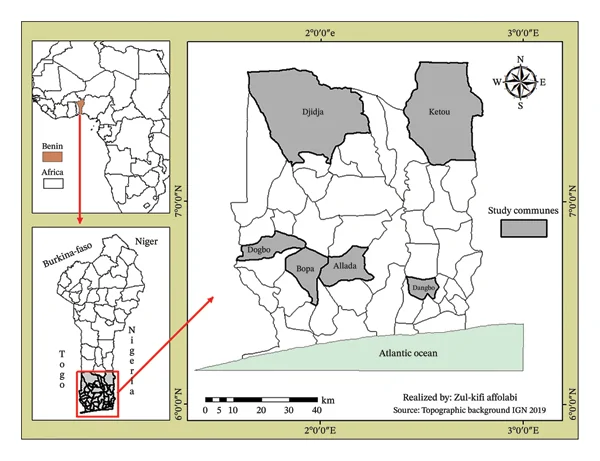
The geographical map of study communes in southern Benin. Map showing the location of the six communes (Allada, Djidja, Bopa, Dangbo, Dogbo, and Ketou) included in the study, spanning the southern departments of Benin where the cross‐sectional survey was conducted (*n* = 384 farmers).

Southern Benin is characterized by a subequatorial climate with two rainy seasons (April–July and October–November) alternating with two dry seasons (December–March and August–September) [24]. The vegetation consists of a mosaic of secondary forests, wooded and shrub savannahs, agricultural landscapes, wetlands, and riparian vegetation, providing suitable habitats for both cyclical and mechanical vectors of animal trypanosomosis [25]. Livestock production in the study area is predominantly based on traditional extensive or semi‐intensive systems. Farmers mainly rear West African Dwarf goats, Djallonké sheep, and local pig breeds under free‐ranging or partially confined management systems with limited veterinary supervision.

### 2.2. Study Design and Sampling

A descriptive cross‐sectional survey was conducted between September and December 2024. The target population consisted of farmers rearing small ruminants (goats, sheep) and/or pigs, aged at least 18 years, with at least 1 year of livestock farming experience, and owning a herd of at least five animals, regardless of species. The minimum required sample size was calculated using Cochran’s formula (*N* = *z* ^2^ × *p* × (1 − *p*)/*e* ^2^) with *z* = 1.96 (95% confidence level), *p* = 0.5 (maximum estimated prevalence in the absence of prior data), and *e* = 0.05, yielding *n* = 384 [26, 27]. Accordingly, 64 farmers were randomly selected per commune from lists provided by local veterinary services. A total of 392 eligible farmers were approached to participate in the survey. Of these, 384 agreed to participate and completed the questionnaire, corresponding to the minimum required sample size and yielding an overall response rate of 98.0%. Eight eligible farmers declined to participate or did not complete the questionnaire and were therefore excluded from the final analysis.

### 2.3. Farming Systems

The surveyed farms were predominantly extensive or semi‐intensive. Herds averaged 7–15 pigs and 8–20 small ruminants per household. Animals typically grazed in free‐ranging systems, supplemented with crop by‐products such as maize, cassava, and oilseed cakes. Access to veterinary services varied across communes and was often limited in remote rural areas.

### 2.4. Data Collection

Data were collected using a structured questionnaire (Supporting File 1) adapted from previously validated KAP survey instruments on tsetse flies and AAT conducted in sub‐Saharan Africa [16, 23]. The questionnaire comprised four sections: (1) respondents’ sociodemographic characteristics; (2) knowledge of tsetse flies and AAT; (3) attitudes toward disease prevention and treatment; and (4) practices related to tsetse fly control and AAT management. Before field implementation, the questionnaire was reviewed by experts in veterinary epidemiology and medical entomology to assess its face validity and was subsequently pretested with 20 livestock farmers who were not included in the final survey. The pilot study evaluated the clarity, comprehensibility, cultural appropriateness, logical sequence, and relevance of the questions, leading to minor revisions in wording and question order to improve administration. Because the questionnaire was designed to collect descriptive information on knowledge, attitudes, and farming practices rather than to measure a single latent construct, formal internal consistency testing was not considered appropriate. Knowledge of tsetse flies and of AAT were each assessed using a single predefined binary (yes/no) item; no composite knowledge score was calculated. The final questionnaire was administered through face‐to‐face interviews by trained enumerators using the KoboCollect mobile application in the respondents’ preferred local language, primarily Fon or Yoruba.

### 2.5. Statistical Analysis

Data were entered into Microsoft Excel and analyzed using R software (Version 4.4.1; R Core Team, Vienna, Austria). Descriptive statistics, including frequencies, percentages, and 95% confidence intervals (CIs), were used to summarize respondents’ sociodemographic characteristics and KAP outcomes. Associations between sociodemographic variables and knowledge of tsetse flies and AAT were assessed using Pearson’s chi‐square (*χ* ^2^) test.

Multivariable logistic regression was used to identify factors independently associated with knowledge of tsetse flies and AAT. Candidate variables (sex, age group, marital status, educational level, occupation, and district) were first screened using bivariable chi‐square tests; variables with *p* < 0.20 were retained and entered simultaneously into the multivariable models. District did not meet this threshold (*p* = 0.462 and *p* = 0.357) and was excluded from the final models. To ensure model validity, multicollinearity among explanatory variables was assessed using the variance inflation factor (VIF), overall model calibration was evaluated using the Hosmer–Lemeshow goodness‐of‐fit test, and model discrimination was assessed by the area under the receiver operating characteristic curve (AUC). Two singleton predictor categories (age 18–20 years; occupation = student) produced quasi‐complete separation were not estimable by maximum likelihood and were therefore excluded from the multivariable models (*n* = 382). Adjusted odds ratios (aORs) and their corresponding 95% 95% CIs were reported. All statistical tests were two‐sided, with statistical significance set a priori at *p* ≤ 0.05.

## 3. Results

### 3.1. Sociodemographic Characteristics of Respondents

Of the 384 livestock farmers surveyed, most were men (62.24%, 95% CI: 57.29–66.94), and nearly half were aged between 41 and 50 years (45.57%, 95% CI: 40.66–50.57), followed by the 31–40 age group (22.40%, 95% CI: 18.51–26.83). The vast majority were married (89.06%, 95% CI: 85.55–91.81); smaller proportions were widowed (5.73%, 95% CI: 3.81–8.52), single (2.86%, 95% CI: 1.61–5.06), or divorced (2.34%, 95% CI: 1.24–4.39). More than half of respondents (56.77%, 95% CI: 51.77–61.63) had received no formal education, while only 4.69% (95% CI: 2.99–7.29) had attained a higher education level. Agriculture (28.65%, 95% CI: 24.35–33.36) and trading (26.04%, 95% CI: 21.90–30.65) were the most common primary occupations, followed by livestock rearing (15.63%, 95% CI: 12.34–19.60); salaried employment (4.43%, 95% CI: 2.78–6.97) and fishing (1.56%, 95% CI: 0.72–3.37) were rare (Figure 2).

**FIGURE 2:**
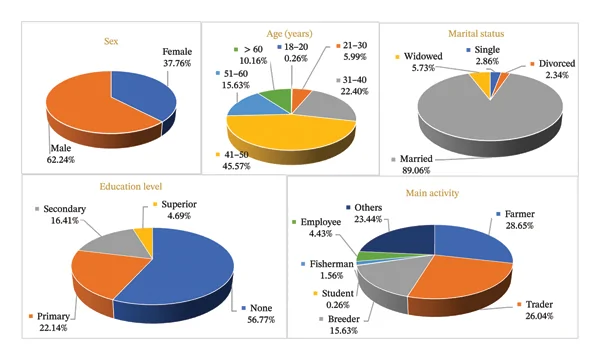
Sociodemographic characteristics of sheep, goat, and pig farmers in the study area. Distribution of respondents (*N* = 384) by sex, age group (years), marital status, educational level, and main occupation. Values are expressed as percentages of the total sample; bars/segments indicate the number (*n*) of farmers in each category.

### 3.2. Knowledge of Tsetse Flies

The majority of farmers (83.59%, 95% CI: 79.51–87.16) were aware of tsetse flies, having usually observed them directly (76.30%, 95% CI: 71.73–80.47); by contrast, veterinarians (3.65%, 95% CI: 2.01–6.04) and other farmers (18.23%, 95% CI: 14.50–22.46) were minor sources of information. Nearly three‐quarters of respondents (73.70%, 95% CI: 68.99–78.03) could identify the areas most affected, chiefly pastures (58.59%, 95% CI: 53.49–63.57), forests (43.49%, 95% CI: 38.47–48.61), and areas near watercourses (36.20%, 95% CI: 31.38–41.23). Tsetse fly abundance was widely perceived to peak during the rainy season (65.63%, 95% CI: 60.64–70.37), with activity concentrated in the morning (58.33%, 95% CI: 53.22–63.31) and evening (50.26%, 95% CI: 45.14–55.37). However, only 16.93% (95% CI: 13.31–21.06) understood that tsetse flies transmit trypanosomosis, even though 77.60% (95% CI: 73.10–81.68) reported having been bitten themselves (Table 1).

**TABLE 1:** Knowledge of tsetse flies among small ruminant and pig farmers in southern Benin (*n* = 384).

Variable	Response	*n* (%)	95% CI
Have you ever heard of or seen tsetse flies?	No	63 (16.41)	12.84–20.50
	Yes	321 (83.59)	79.51–87.16

Main source of information^∗^	Personal observation	293 (76.30)	71.73–80.47
	Other farmers	70 (18.23)	14.50–22.46
	Veterinarian	14 (3.65)	2.01–6.04
	Training	1 (0.26)	0.01–1.44

Do you know the areas most affected by tsetse flies in your region?	No	38 (9.90)	7.10–13.33
	Yes	283 (73.70)	68.99–78.03

Areas where tsetse flies are most common^∗^	Pastures	225 (58.59)	53.49–63.57
	Forests	167 (43.49)	38.47–48.61
	Near water bodies	139 (36.20)	31.38–41.23
	Animal shelters	3 (0.78)	0.16–2.27

Season of highest tsetse abundance	Rainy season	252 (65.63)	60.64–70.37
	Dry season	9 (2.34)	1.08–4.40
	Throughout the year	18 (4.69)	2.80–7.31
	No opinion	42 (10.94)	8.00–14.50

Time of day when tsetse flies are most active^∗^	Morning	224 (58.33)	53.22–63.31
	Evening	193 (50.26)	45.14–55.37
	Afternoon	85 (22.14)	18.08–26.63
	At any time	11 (2.86)	1.44–5.07
	No opinion	74 (19.27)	15.45–23.58

Did you know that tsetse flies transmit trypanosomosis?	No	256 (66.67)	61.71–71.37
	Yes	65 (16.93)	13.31–21.06

Mode of transmission	Through the bite (injection)	65 (16.93)	13.31–21.06

Have you or a family member ever been bitten by a tsetse fly?	No	23 (5.99)	3.83–8.85
	Yes	298 (77.60)	73.10–81.68

*Note:* Values are presented as number (percentage) with corresponding 95% confidence interval (95% CI).

^∗^Multiple responses were allowed.

### 3.3. Knowledge and Perceptions of AAT

Awareness of AAT itself was considerably lower: 81.25% (95% CI: 76.98–85.03) of farmers had never heard of the disease, and only 18.75% (95% CI: 14.97–23.02) had. Among those who had, veterinarians were the main source of information (10.94%, 95% CI: 8.00–14.50), with radio, training, and neighbors each cited by fewer than 3% of respondents. Few farmers regarded AAT as a major problem (16.41%, 95% CI: 12.84–20.50), and even fewer described its impact on livestock production as strong (9.64%, 95% CI: 6.88–13.04) or very strong (6.77%, 95% CI: 4.47–9.76). As with tsetse fly abundance, the rainy season was most often identified as the period of highest AAT occurrence (13.54%, 95% CI: 10.28–17.38) (Figure 3; Table 2).

**FIGURE 3:**
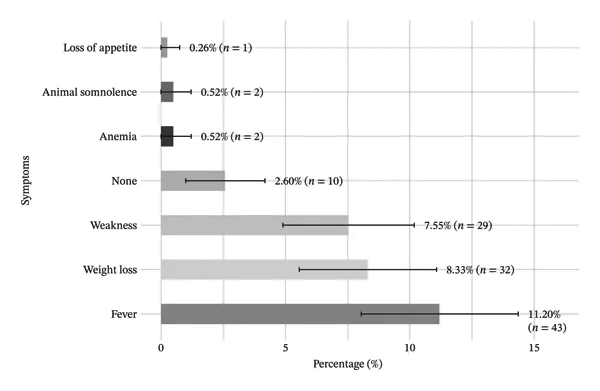
Animal trypanosomosis symptoms and clinical signs mentioned by the farmers. Frequency of clinical signs of Animal African trypanosomosis (AAT) reported by respondents (*N* = 384): fever, weight loss, weakness, anemia, somnolence, loss of appetite, or no symptoms reported (“None”). Values are percentages of the total sample with 95% Wilson confidence intervals (error bars); farmers could report more than one symptom.

**TABLE 2:** Knowledge and perceptions of Animal African trypanosomosis among small ruminant and pig farmers in southern Benin (*n* = 384).

Variable	Response	*n* (%)	95% CI
Do you know about Animal African Trypanosomosis (AAT)?	No	312 (81.25)	76.98–85.03
	Yes	72 (18.75)	14.97–23.02

Main source of information	Veterinarians	42 (10.94)	8.00–14.50
	Radio	8 (2.08)	0.90–4.06
	Training	8 (2.08)	0.90–4.06
	Neighbors	4 (1.04)	0.29–2.65
	Personal observation	2 (0.52)	0.01–1.87
	No response	10 (2.60)	1.26–4.74

Do you consider AAT a major problem in your area?	Yes	63 (16.41)	12.84–20.50
	I don’t know	9 (2.34)	1.08–4.40

Impact of AAT on livestock production	Strong	37 (9.64)	6.88–13.04
	Very strong	26 (6.77)	4.47–9.76
	I don’t know	9 (2.34)	1.08–4.40

Season when AAT is most frequent	Rainy season	52 (13.54)	10.28–17.38
	Dry season	1 (0.26)	0.01–1.44
	No opinion	19 (4.95)	3.01–7.62

*Note:* Values are presented as number (percentage) with corresponding 95% confidence intervals (95% CI).

### 3.4. Attitudes and Practices Regarding Tsetse Fly Control

Insecticides were the method most frequently regarded as effective against tsetse flies (44.53%, 95% CI: 39.49–49.66), although more than a third of respondents (35.94%, 95% CI: 31.13–40.96) had no opinion on the matter. Perceptions of the importance of tsetse fly control for preventing trypanosomosis were similarly divided: Only 21.88% (95% CI: 17.84–26.35) considered it essential, while 37.24% (95% CI: 32.39–42.29) expressed no view. Despite this, most farmers (66.41%, 95% CI: 61.44–71.12) reported using no specific control method. Among those who did, insecticides again predominated (14.06%, 95% CI: 10.74–17.95), followed by traditional plant‐based methods (2.86%, 95% CI: 1.44–5.07), smoke (1.04%, 95% CI: 0.29–2.65), and manual destruction (0.78%, 95% CI: 0.16–2.27). The plants most commonly cited were *Hyptis suaveolens* and *Eucalyptus* spp. (each 1.56%, 95% CI: 0.58–3.37), followed by neem (0.26%, 95% CI: 0.01–1.44). Method selection was primarily driven by perceived effectiveness (15.36%, 95% CI: 11.91–19.37), followed by availability (12.24%, 95% CI: 9.13–15.94) and low cost (9.90%, 95% CI: 7.10–13.33) (Table 3).

**TABLE 3:** Farmers’ attitudes and practices regarding tsetse fly control among small ruminant and pig farmers in southern Benin (*n* = 384).

Variable	Response	*n* (%)	95% CI
Perceived best method for tsetse fly control	Insecticide use	171 (44.53)	39.49–49.66
	Vegetation clearing	19 (4.95)	3.01–7.62
	Improved livestock management	22 (5.73)	3.63–8.55
	Smoke	8 (2.08)	0.90–4.06
	No opinion	138 (35.94)	31.13–40.96

Importance of tsetse fly control for preventing AAT	Important	84 (21.88)	17.84–26.35
	Moderately important	1 (0.26)	0.01–1.44
	Very important	93 (24.22)	20.02–28.82
	No opinion	143 (37.24)	32.39–42.29

Reducing the tsetse fly population is essential to protect livestock	Yes	187 (48.70)	43.60–53.82
	No	134 (34.90)	30.13–39.90

Current use of tsetse fly control methods	Yes	66 (17.19)	13.55–21.34
	No	255 (66.41)	61.44–71.12

Control methods currently used^∗^	Insecticides	54 (14.06)	10.74–17.95
	Traps	1 (0.26)	0.01–1.44
	Vegetation clearing	1 (0.26)	0.01–1.44
	Traditional plant‐based methods	11 (2.86)	1.44–5.07
	Mechanical killing	3 (0.78)	0.16–2.27
	Smoke	4 (1.04)	0.29–2.65
	Diesel/kerosene	3 (0.78)	0.16–2.27

Plant species used^∗^	*Azadirachta indica* (Neem)	1 (0.26)	0.01–1.44
	*Eucalyptus* spp.	6 (1.56)	0.58–3.37
	*Hyptis suaveolens*	6 (1.56)	0.58–3.37

Reasons for choosing these methods^∗^	Perceived effectiveness	59 (15.36)	11.91–19.37
	Availability	47 (12.24)	9.13–15.94
	Low cost	38 (9.90)	7.10–13.33

*Note:* Values are presented as the number (percentage) with corresponding 95% confidence intervals (95% CI). Percentages were calculated using the total study population (*n* = 384).

^∗^Multiple responses were allowed.

### 3.5. Prevention and Treatment Practices for AAT

Among the 384 farmers, few reported taking preventive action against AAT: 10.68% (95% CI: 7.97–14.16) administered trypanocides and 5.73% (95% CI: 3.81–8.52) controlled tsetse flies specifically, while 4.95% (95% CI: 3.19–7.60) took no preventive measures at all (Figure 4). When treatment was required, trypanocides were the predominant choice (11.46%, 95% CI: 8.45–15.08), with plant‐based remedies, chiefly *Azadirachta indica* (neem) and *Moringa oleifera* leaves, used far less often (0.52%, 95% CI: 0.06–1.87). Veterinary pharmacies were the main source of medication (10.42%, 95% CI: 7.55–13.91), and treatment was more often regular (5.73%, 95% CI: 3.63–8.55) than occasional (4.17%, 95% CI: 2.40–6.68) or reserved for suspected cases (0.52%, 95% CI: 0.06–1.87). Veterinarians administered most treatments (9.38%, 95% CI: 6.65–12.74), whereas farmer self‐medication was uncommon (2.34%, 95% CI: 1.08–4.40). Veterinary services were widely used overall (82.29%, 95% CI: 78.10–85.98), although consultations were driven mainly by suspected illness (71.35%, 95% CI: 66.57–75.74) rather than routine visits (25.00%, 95% CI: 20.76–29.64). Notably, veterinarians did not always explain the targeted disease to farmers (63.54%, 95% CI: 58.52–68.34), pointing to a communication gap in veterinary care delivery (Table 4).

**FIGURE 4:**
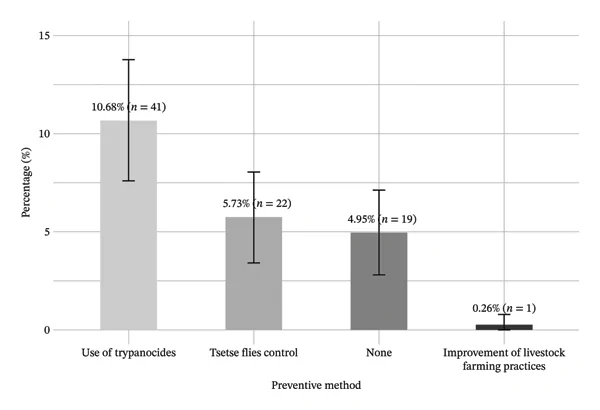
Preventive methods against AAT reported by farmers. Distribution of preventive measures against Animal African trypanosomosis reported by respondents (*N* = 384): use of trypanocides, tsetse fly control, improvement of livestock farming practices, or no measure applied (“None”). Values are percentages of the total sample with 95% Wilson confidence intervals (error bars).

**TABLE 4:** Farmers’ attitudes and practices regarding the prevention and treatment of Animal African trypanosomosis among small ruminant and pig farmers in southern Benin (*n* = 384).

Variable	Response	*n* (%)	95% CI
Treatment used for Animal African Trypanosomosis	Trypanocides	44 (11.46)	8.45–15.08
	Traditional plant‐based treatment	2 (0.52)	0.06–1.87

Traditional remedies used^∗^	*Azadirachta indica* leaves	1 (0.26)	0.01–1.44
	*Moringa oleifera* leaves	1 (0.26)	0.01–1.44

Source of treatment	Veterinary pharmacy	40 (10.42)	7.55–13.91
	Government veterinary services	2 (0.52)	0.06–1.87

Frequency of treatment	Occasionally	16 (4.17)	2.40–6.68
	Regularly	22 (5.73)	3.63–8.55
	Only when disease is suspected	2 (0.52)	0.06–1.87

Person administering treatment^∗^	Farmer	9 (2.34)	1.08–4.40
	Veterinarian	36 (9.38)	6.65–12.74

Use of veterinary services	Yes	316 (82.29)	78.10–85.98
	No	68 (17.71)	14.02–21.90

Frequency of veterinary consultation	When disease is suspected	274 (71.35)	66.57–75.74
	Routine visits	96 (25.00)	20.76–29.64

Do veterinarians explain the diseases targeted by treatment?	Yes	72 (18.75)	14.97–23.02
	No	244 (63.54)	58.52–68.34

*Note:* Values are presented as number (percentage) with corresponding 95% confidence interval (95% CI). Percentages were calculated using the total study population (*n* = 384).

^∗^Multiple responses were allowed where applicable.

### 3.6. Factors Associated With Farmers’ Knowledge

Knowledge of tsetse flies and AAT varied significantly across sociodemographic groups (Table 5). Men were consistently better informed than women (*χ* ^2^ = 74.53, *p* < 0.001 for tsetse flies; *χ* ^2^ = 33.85, *p* < 0.001 for AAT), and knowledge increased markedly with age, peaking in the 41–50‐year group (*χ* ^2^ = 299.04 and 83.79, respectively; both *p* < 0.001). Married individuals were the most informed (*χ* ^2^ = 873.37 and *χ* ^2^ = 169.76; both *p* < 0.001), and educational attainment was similarly influential, with farmers holding secondary or higher education showing the greatest awareness (*χ* ^2^ = 219.96, *p* < 0.001 for tsetse flies; *χ* ^2^ = 22.27, *p* < 0.001 for AAT). Occupation was also associated with knowledge, particularly of tsetse flies (*χ* ^2^ = 214.23, *p* < 0.001; AAT: *χ* ^2^ = 45.10, *p* < 0.001). Commune of residence, by contrast, showed no significant association with knowledge of either tsetse flies (*χ* ^2^ = 4.64, *p* = 0.462) or AAT (*χ* ^2^ = 5.51, *p* = 0.357) and was therefore excluded from the multivariable models.

**TABLE 5:** Bivariable associations between sociodemographic characteristics and knowledge of tsetse flies and Animal African trypanosomosis among small ruminant and pig farmers in southern Benin (*n* = 384).

Variable	Category	Knowledge of tsetse flies, *n* (%)	*χ* ^2^	*p* value	Knowledge of AAT, *n* (%)	*χ* ^2^	*p* value
Sex	Female	101 (26.30)	74.53	< 0.001	12 (3.13)	33.85	< 0.001
	Male	220 (57.29)			60 (15.63)		

Age group (years)	18–20	1 (0.26)	299.04	< 0.001	0 (0.00)	83.79	< 0.001
	21–30	19 (4.95)			8 (2.08)		
	31–40	66 (17.19)			12 (3.13)		
	41–50	150 (39.06)			39 (10.16)		
	51–60	50 (13.02)			10 (2.60)		
	> 60	35 (9.11)			3 (0.78)		

Marital status	Single	9 (2.34)	873.37	< 0.001	1 (0.26)	169.76	< 0.001
	Divorced	8 (2.08)			0 (0.00)		
	Married	284 (73.96)			59 (15.36)		
	Widowed	20 (5.21)			2 (0.52)		

Educational level	None	177 (46.09)	219.96	< 0.001	22 (5.73)	22.27	< 0.001
	Primary	68 (17.71)			11 (2.86)		
	Secondary	59 (15.36)			32 (8.33)		
	Higher	17 (4.43)			7 (1.82)		

Main occupation	Farmer	98 (25.52)	214.23	< 0.001	22 (5.73)	45.10	< 0.001
	Trader	72 (18.75)			13 (3.39)		
	Livestock farmer	57 (14.84)			21 (5.47)		
	Student	1 (0.26)			0 (0.00)		
	Fisher	6 (1.56)			3 (0.78)		
	Employee	16 (4.17)			5 (1.30)		
	Other	71 (18.49)			8 (2.08)		

Commune	Allada	49 (12.76)	4.64	0.462	17 (4.43)	5.51	0.357
	Djidja	61 (15.89)			15 (3.91)		
	Bopa	56 (14.58)			13 (3.39)		
	Dangbo	43 (11.20)			10 (2.60)		
	Dogbo	54 (14.06)			8 (2.08)		
	Kétou	58 (15.10)			9 (2.34)		

*Note:* Values are presented as number (percentage). Associations between sociodemographic characteristics and knowledge of tsetse flies or Animal African trypanosomosis (AAT) were assessed using Pearson’s chi‐square test. Statistical significance was set at *p* < 0.05. Percentages were calculated using the total study population (*n* = 384).

In the multivariable analysis (Table 6), secondary and higher education remained strong independent predictors of AAT knowledge (aOR = 7.21, 95% CI: 3.43–15.54, *p* < 0.001; aOR = 5.52, 95% CI: 1.30–25.03, *p* = 0.022, respectively). Male sex was similarly associated with greater odds of knowledge, for both tsetse flies (aOR = 4.45, 95% CI: 2.10–9.78, *p* < 0.001) and AAT (aOR = 3.35, 95% CI: 1.37–8.71, *p* = 0.009). Occupation in agriculture (aOR = 3.48, 95% CI: 1.33–9.85, *p* = 0.014) or livestock rearing (aOR = 6.52, 95% CI: 2.47–18.63, *p* < 0.001) further predicted AAT knowledge, whereas age and marital status showed no significant association after adjustment (all *p* > 0.05).

**TABLE 6:** Multivariable logistic regression analysis of factors associated with knowledge of tsetse flies and Animal African trypanosomosis among small ruminant and pig farmers in southern Benin (*n* = 384).

Variable	Category	Knowledge of tsetse flies aOR (95% CI)	*p* value	Knowledge of AAT aOR (95% CI)	*p* value
Sex	Female (reference)	—	—	—	—
	Male	4.45 (2.10–9.78)	< 0.001	3.35 (1.37–8.71)	0.009

Age group (years)	21–30 (reference)	—	—	—	—
	31–40	1.06 (0.19–4.87)	0.939	0.45 (0.10–2.05)	0.300
	41–50	1.81 (0.32–8.09)	0.461	0.72 (0.18–3.05)	0.654
	51–60	1.28 (0.20–6.80)	0.775	0.47 (0.09–2.40)	0.360
	> 60	1.30 (0.17–9.05)	0.792	0.21 (0.03–1.38)	0.116

Marital status	Single (reference)	—	—	—	—
	Married	1.17 (0.11–9.27)	0.888	7.18 (0.67–182.97)	0.142
	Widowed	4.20 (0.26–69.89)	0.302	16.41 (0.74–589.94)	0.084
	Divorced	1.51 (0.07–50.25)	0.793	—	—

Educational level	No formal education (reference)	—	—	—	—
	Primary	0.56 (0.26–1.23)	0.146	1.32 (0.55–3.04)	0.529
	Secondary	2.16 (0.75–7.89)	0.189	7.21 (3.43–15.54)	< 0.001
	Higher	1.16 (0.13–27.82)	0.908	5.52 (1.30–25.03)	0.022

Occupation	Other (reference)	—	—	—	—
	Farmer	1.54 (0.60–3.99)	0.368	3.48 (1.33–9.85)	0.014
	Trader	0.95 (0.37–2.41)	0.914	3.83 (1.21–12.75)	0.024
	Livestock farmer	3.80 (1.06–18.45)	0.059	6.52 (2.47–18.63)	< 0.001
	Fisher	—	—	6.63 (0.84–50.98)	0.063
	Employee	1.73 (0.19–41.78)	0.600	1.61 (0.30–8.04)	0.564

*Note:* Reference categories are shown in parentheses. Variables with *p* < 0.20 in the bivariable analyses were entered into the multivariable logistic regression model. *p* < 0.05 was considered statistically significant.

Abbreviations: aOR, adjusted odds ratio; CI, confidence interval.

Based on 382 valid observations (two singleton categories excluded), all VIFs were below 10, the commonly accepted threshold for problematic multicollinearity; age (31–40, 41–50 years) and marital status (married) showed moderately elevated values (VIF 5–7). Discriminative performance was good, with an AUC of 0.77 for the tsetse fly knowledge model and 0.82 for the AAT knowledge model; McFadden’s pseudo‐*R* ^2^ was 0.15 and 0.23, and Nagelkerke’s pseudo‐*R* ^2^ approximately 0.21 and 0.32, respectively. The Hosmer–Lemeshow test indicated adequate calibration for the AAT model (*p* > 0.05) but suggested some lack of fit for the tsetse fly model (*p* < 0.05). Quasi‐complete separation was observed for two categories, fisherman (*n* = 6) in the tsetse fly model and divorced (*n* = 9) in the AAT model.

## 4. Discussion

This study provides the first comprehensive KAP assessment of rural small ruminant and pig farmers regarding tsetse flies and AAT in southern Benin. The findings reveal a marked discrepancy between farmers’ ability to recognize tsetse flies and their understanding of the fly’s epidemiological role in AAT transmission, suggesting that routine vector exposure does not translate into effective disease knowledge or preventive practices.

The high recognition of tsetse flies is consistent with recent reports from Kenya and Ethiopia, where livestock keepers identify the fly by its distinctive morphology and painful bite rather than through formal training [28, 29]. In the Kenyan study around the Arabuko Sokoke Forest Reserve, 98% of respondents recognized the tsetse fly, yet only 16% could identify it as a disease vector [28], a pattern strikingly similar to the recognition–knowledge gap observed here. A comparable dissociation between the exposure‐based recognition of a hazard and understanding of its epidemiological role has also been documented among actors in the livestock trade confronting other zoonotic and vector‐borne diseases in East Africa [30], and among cattle farmers assessing tick‐borne disease risk in Nigeria [31]. Recognition of vectors therefore likely reflects occupational exposure in grazing environments rather than sound vector‐biology understanding and cannot be considered a reliable AAT‐preparedness indicator.

Knowledge of AAT itself was strikingly low here, echoing Kenya, where adequate combined knowledge was found in only 16% of farmers [28]. Substantially higher awareness has been reported where veterinary extension and community‐based tsetse control are sustained, as in northern Benin, where 84% of cattle farmers recognized tsetse flies as AAT vectors [16], and in central Ethiopia, where two‐thirds of farmers correctly identified the vector [29]. AAT nonetheless remains epidemiologically significant regionally: A recent meta‐analysis of West African cattle confirmed the persistence of highly endemic pockets despite decades of control programs [9], and a systematic review in Uganda similarly documented substantial and geographically uneven AAT prevalence across eight host species [32]. These differences underline the importance of continuous farmer education in improving disease awareness and promoting evidence‐based livestock management.

Several interacting factors probably explain the limited awareness observed among respondents. More than half of the farmers had no formal education, restricting access to written information and reducing comprehension of prevention messages, while veterinary extension remains insufficient in rural Benin. Control initiatives have historically targeted cattle for their greater economic importance, leaving small ruminant and pig farmers underserved, a gap also noted in the only other published Beninese AAT‐KAP survey, which focused exclusively on cattle owners [16]. Furthermore, where tsetse populations have declined, farmers may perceive trypanosomosis as a less immediate threat, illustrated by the recent AAT elimination from Senegal’s Niayes livestock zone following area‐wide tsetse eradication combining insecticide‐based control with the sterile insect technique [33]. Finally, the elimination of HAT as a public health problem in Benin, validated by WHO in 2022 [11, 34], may have unintentionally diminished public attention to the continued endemicity of AAT.

Educational attainment emerged as one of the strongest independent predictors of farmers’ knowledge, consistent with Kenya, where secondary and tertiary education were associated with 2.5‐fold and 3.8‐fold higher odds of adequate knowledge compared with no formal education [28] and with coastal Ghana, where formal education was likewise the strongest correlate of good zoonotic‐disease knowledge [35]. This probably reflects greater access to veterinary information, improved health literacy, and enhanced capacity to interpret disease prevention messages. These findings emphasize the need to adapt extension strategies to the educational background of livestock farmers by promoting practical demonstrations, pictorial educational materials, local‐language communication, and community‐based awareness campaigns.

Male farmers also demonstrated significantly greater knowledge than female respondents, plausibly reflecting men’s greater involvement in animal marketing and veterinary consultations; a similar, nonsignificant trend was reported among Kenyan livestock keepers [28]. As women increasingly contribute to small ruminant and pig production in West Africa, gender‐sensitive extension programs ensuring equitable access to animal health information are needed, as similarly recommended following a Cameroonian small ruminant KAP survey [36].

Despite recognizing insecticides as the most effective method for controlling tsetse flies, relatively few farmers reported using them, a knowledge–practice gap also documented in northern Benin, where only 24% of AAT‐aware farmers considered insecticide‐based vector control effective despite near‐universal reliance on curative trypanocides [16]. A prospective cohort study comparing four control strategies in Eastern Zambia confirmed that insecticide‐based interventions reduced AAT incidence more effectively than curative trypanocide treatment alone, yet remain markedly under‐used relative to chemotherapy [37]. Financial constraints, limited product availability, safety concerns, and inadequate guidance likely contribute to this low uptake, and addressing them will require improved awareness alongside greater access to affordable tools.

Preventive practices against AAT were similarly inadequate, with most respondents relying on treatment only after clinical signs appeared. Such reactive approaches may contribute to continued parasite circulation, delayed diagnosis, inappropriate trypanocide use, and drug resistance, a pattern confirmed by a systematic review and meta‐analysis identifying poor farmer knowledge and self‐administration as major resistance drivers [38], corroborated by a study of trypanocide usage in southwestern Uganda’s cattle belt [39] and by a longitudinal investigation of trypanocide failure in cattle near northern Tanzania’s wildlife areas [40]. The economic consequences are considerable: Household‐level studies in Ethiopia and Uganda documented substantial annual expenditure on trypanocidal drugs and losses linked to mortality and reduced draught power [41, 42]. Strengthening farmer education on preventive herd health management should therefore accompany improved access to veterinary services.

From a One Health perspective, integrating veterinary extension, vector surveillance, environmental management, and community participation remains essential. Although HAT was validated by WHO as eliminated as a public health problem in Benin in 2022 [11, 34], the continued circulation of animal trypanosomes and their vectors justifies maintaining surveillance at the animal–vector–environment interface. The Senegalese experience demonstrates that, where sustained investment and community engagement combine with area‐wide vector control, the elimination of AAT from a livestock production zone is achievable [33].

This study’s strengths include being the first investigation specifically targeting small ruminant and pig farmers across southern Benin while applying multivariable logistic regression to identify independent predictors of knowledge, an approach comparable to recent AAT‐KAP studies elsewhere on the continent [28]. Limitations include the cross‐sectional design, which precludes causal inference; self‐reported practices subject to recall and social desirability biases, a limitation also flagged in comparable recent surveys [29]; the absence of a validated composite knowledge score; and some imprecision in aORs despite acceptable‐to‐good model discrimination (AUC = 0.77 and 0.82) and imperfect calibration for the tsetse fly knowledge model (Hosmer–Lemeshow *p* < 0.05), likely reflecting outcome imbalance and sparse observations in subgroups. In addition, the exclusive use of quantitative methods is a limitation, as qualitative approaches such as focus‐group discussions or in‐depth interviews could have provided a deeper understanding of farmers’ perceptions and behavioral determinants underlying their KAP. Larger, mixed‐methods studies combining quantitative surveys with qualitative inquiry would help refine these estimates and better capture the sociobehavioral drivers of AAT‐related practices.

Overall, the findings support strengthening veterinary extension services and developing targeted educational programs on tsetse identification, disease transmission, and integrated vector management, alongside improved access to veterinary services, as recommended in comparable settings in Ethiopia and Benin [16, 29]. These interventions should be integrated within a One Health framework involving veterinary, agricultural, and environmental sectors to improve livestock productivity while reducing the burden of vector‐borne diseases in Benin and beyond.

## 5. Conclusions

This study provides the first comprehensive assessment of the KAP of small ruminant and pig farmers regarding tsetse flies and AAT in southern Benin. Although most farmers were able to recognize tsetse flies, awareness of AAT, its transmission, clinical manifestations, and effective prevention measures remained markedly limited. The low adoption of vector control practices and preventive herd health measures further highlights important gaps that may contribute to the persistence of disease transmission in smallholder livestock production systems.

Educational attainment, sex, and occupation were significant determinants of disease awareness, indicating that socioeconomic factors strongly influence farmers’ access to animal health information. These findings emphasize the need for targeted veterinary extension programs specifically designed for small ruminant and pig farmers, who have historically received less attention than cattle producers in national trypanosomosis control initiatives.

Strengthening farmer education, expanding access to veterinary advisory services, promoting community‐based vector management, and integrating AAT into broader One Health programs should be considered priorities for improving livestock health and productivity in Benin. Future studies combining quantitative and qualitative approaches are warranted to better understand behavioral barriers to the adoption of recommended prevention and control measures and to guide the development of sustainable, evidence‐based intervention strategies.

**Nomenclature**
AAT: Animal African trypanosomosis
CI: Confidence interval
HAT: Human African trypanosomiasis
KAP: Knowledge, attitudes, and practices
OR: Odds ratio
WHO: World Health Organization

## Author Contributions

The study was designed and coordinated by Zul‐Kifl Affolabi and Razaki A. Ossè. Razaki A. Ossè provided resources. Data collection was performed by Zul‐Kifl Affolabi, Koffi Djigbodi Koumodji, Minassou Juvénal Ahouandjinou, and Steve Zinsou Hougbe. Zul‐Kifl Affolabi drafted the manuscript. Zul‐Kifl Affolabi and Ehouzou Olivier Oussou performed the statistical analysis. Razaki A. Ossè, Prudenciène A. Agboho, Côme Zinsou Koukpo, Arsène Jacques Fassinou, Casimir Kpanou, Filémon Tokponnon, Germain Gil Padonou, and Martin Akogbeto revised the manuscript.

## Funding

This research received no external funding.

## Disclosure

The corresponding author affirms that this manuscript is an honest, accurate, and transparent account of the study being reported, that no important aspects of the study have been omitted, and that any discrepancies from the study as originally planned have been explained. All authors read and approved the final manuscript. The corresponding author had full access to all study data and takes full responsibility for the integrity of the data and the accuracy of the data analysis.

## Ethics Statement

The study protocol was reviewed and approved by the Institutional Ethics Committee for Health Research of the Centre de Recherche Entomologique de Cotonou (CIERS‐CREC). Free and informed consent was obtained from each participant following clear presentation of the study’s objectives. Anonymity and confidentiality of all data were strictly maintained.

## Consent

Please see the Ethics Statement.

## Conflicts of Interest

The authors declare no conflicts of interest.

## Supporting Information

Additional supporting information can be found online in the Supporting Information section.

## Supporting information


**Supporting Information** Supporting File 1: Survey questionnaire used in the study.

## Data Availability

The datasets generated and analyzed during the current study are available from the corresponding author upon reasonable request.
