# Supplementary material for: Knowledge, Attitudes, and Practices of Small Ruminant and Pig Farmers Regarding Tsetse Flies and Animal African Trypanosomosis in Southern Benin
Source: Vet Med Int. 2026 Sep 30;2026:8578242. doi: 10.1155/vmi/8578242 (PMC13628160; doi:10.1155/vmi/8578242)
Supplement: Supplementary file 1 [file VMI-2026-8578242-s001.pdf]

Survey form on knowledge, attitudes and practices of livestock farmers on animal trypanosomiasis and its vectors

**Details of the survey location**			
**Country :**	**Department :**	**Commune/District:**	**Village/Neighborhood:** ....

I. SOCIO-DEMOGRAPHIC CHARACTERISTICS

Name and first name of the breeder (surveyed) :...

I.1. Gender Male ☐ Female ☐

I.2. Age 18-20 ☐ 21-30 ☐ 31-40 ☐ 41-50 ☐ 51-60 ☐ >60 ☐

I.3. Marital status

☐ Single Married ☐ Widowed ☐ Divorced ☐

I.4. Level of education

No ☐ ☐ Primary ☐ Secondary ☐ Higher

☐ Others (please specify):...

I.5. Main activities

☐ Farmer ☐ Breeder ☐ Trader ☐ Employee

Fisherman ☐ ☐ Other (to be specified) ....

I.6. Types of domestic animals raised

Sheep ☐ Cattle ☐ Goats ☐ Pigs ☐

I.7. Livestock size

<10 ☐ 10 to 20 ☐ 21 to 30 ☐ 31 to 40 ☐ 41 to 50 ☐ >50 ☐

1.8. Geographic location of the farm (observe for information)

Near a stream ☐ **Forest** ☐ **Savannah** ☐

☐ **Others (specify) :**...

II. Knowledge of tsetse flies and animal trypanosomiasis

II.1 Knowledge of tsetse flies

Have you ever heard of or seen tsetse flies?

Yes ☐ No ☐

If yes, what is your main source of information? (check all that apply)

Radio ☐ Television ☐ Newspapers ☐ Internet ☐ Other farmers/ ranchers ☐

Vocational training ☐ Veterinary health agents ☐ Personal observation ☐

☐ Others (specify) :..

....

Do you know the areas most affected by tsetse flies in your region?

Yes ☐ No ☐

If yes, please specify:

Near watercourses ☐ Pastures ☐ Forests ☐

In the enclosures ☐ ☐ Other (specify) :...

When are these flies most abundant during the year?

Rainy season ☐ Dry season ☐ All year round ☐ No idea ☐

What time of day are they most active?

Morning ☐ Afternoon ☐ Evening ☐ Night ☐ Anytime ☐

No idea ☐

Did you know that tsetse flies transmit trypanosomiasis to your animals?

Yes ☐ No ☐

If so, how do tsetse flies transmit animal trypanosomiasis?

By sting ☐ By direct contact ☐ By water ☐ I don't know ☐

Have you or a family member ever been bitten by these flies?

Yes ☐ No ☐

II.2. Knowledge of the symptoms of the disease

What **are the symptoms of animal trypanosomiasis that you are familiar with? (check all that apply)**

Fever ☐ Anemia ☐ Weight loss ☐ Weakness ☐

☐ Other (specify) :………

II. 3. Sources of information

Where do you usually get your information on tsetse and animal trypanosomiasis?

Radio ☐ Training ☐ Neighbors ☐ Veterinarians ☐ Internet ☐

☐ Others (specify):……

Have you attended any training or workshops on this topic?

☐ **Yes** ☐ **No**

III. Attitudes Toward Prevention and Treatment

III.1 Perception of gravity

Do you think animal trypanosomiasis is a major problem in your area?

Yes ☐ No ☐ I don't know ☐

To what extent does animal trypanosomiasis affect your livestock farming business?

Very strongly ☐ Strongly ☐ Moderately ☐ Little ☐ Not at all ☐

Have you noticed times of the year when trypanosomiasis is more common?

Yes ☐ ☐ No

If yes, please specify

………

III.2 Perception and attitudes towards control methods

What do you think is the best way to control tsetse flies?

Use of insecticides ☐ Clearing of vegetation ☐ Use of tsetse traps ☐

Improvement of breeding conditions ☐

☐ Other (specify) :……

Are you ready to participate in tsetse control programs in your area?

Yes ☐ No ☐ Maybe ☐

III.3 Beliefs about the importance of tsetse control

How important is the fight against tsetse flies to you in preventing animal trypanosomiasis?

Very important ☐ Important ☐ Moderately important ☐

Not important ☐ Not at all important ☐

IV. Tsetse and trypanosomiasis management and control practices

IV.1 Control methods

Do you use any methods to control tsetse flies on your farm?

☐ Yes ☐ No

If yes, what methods do **you use? (Check all that apply)**

Modern insecticides ☐ Traps ☐ Insecticide - impregnated screens ☐

Vegetation clearing ☐ Traditional methods (plants) ☐

☐ Other (specify): ………

………

If there are uses for plants, what are they?

Basil ☐ Lemongrass ☐ Neem ☐ Eucalyptus ☐

☐ Others: **……………………………………………………………………………………………………**

In what forms do you use these plants?

Fumigation ☐ Decoction ☐ Maceration in water ☐ Maceration in ethanol ☐

Freshly ground plants ☐ Herbal powders ☐

Hanging insect repellent plants in enclosures ☐ Planting repellent plants ☐

Others :……

……

Why did you choose these specific methods?

Cost ☐ Availability ☐ Perceived effectiveness ☐

Others (specify) :……

……

IV.2 Prevention and treatment practices

What measures do you take to prevent trypanosomiasis in your animals?

Use of trypanocides ☐ Control of tsetse flies ☐

Use of repellents and nets ☐ Improvement of breeding practices ☐

Others (specify) ……

……

What treatments do you use when your animals have trypanosomiasis?

Trypanocides ☐ Use of plants ☐

Others (specify) ………

……….

Where do you get trypanocides for trypanosomiasis?

Veterinary pharmacy ☐ Government veterinary services ☐ Private provider ☐

Other (specify): ……………………………………………………………………………………………………

……. ☐ ……….

How often do you apply these control methods and treatments?

Never ☐ Occasion ☐ Regularly ☐

Only in case of suspected disease ☐

Who is responsible for applying these methods on your farm?

Yourself ☐ A family member ☐ A veterinarian ☐

Others (specify)……….

IV.3 Usual treatment practices

Do you often use the services of a veterinarian?

Yes ☐ No ☐

In which cases?

According to a well-defined schedule ☐ Only in case of suspected disease ☐

if , treatments according to a well-defined schedule, specify how many times a year?

Once a year ☐ Twice a year ☐ Three times a year ☐

Does not know ☐

Does the veterinarian explain to you the diseases for which the treatments are administered?

Yes ☐ No ☐
